# Sifalimumab, a Human Anti–Interferon-α Monoclonal Antibody, in Systemic Lupus Erythematosus: A Phase I Randomized, Controlled, Dose-Escalation Study

**DOI:** 10.1002/art.37824

**Published:** 2013-03-28

**Authors:** Michelle Petri, Daniel J Wallace, Alberto Spindler, Vishala Chindalore, Kenneth Kalunian, Eduardo Mysler, C Michael Neuwelt, Gabriel Robbie, Wendy I White, Brandon W Higgs, Yihong Yao, Liangwei Wang, Dominique Ethgen, Warren Greth

**Affiliations:** 1 Johns Hopkins University School of MedicineBaltimore, Maryland; 2 Cedars-Sinai Medical Center and David Geffen School of Medicine, University of CaliforniaLos Angeles; 3 Centro Medico Privado de ReumatologiaTucumán, Argentina; 4 Pinnacle Research GroupAnniston, Alabama; 5 University of California at San DiegoLa Jolla; 6 Organización Médica de InvestigaciónBuenos Aires, Argentina; 7 East Bay Rheumatology Research InstituteSan Leandro, California; 8 MedImmune, LLCGaithersburg, Maryland

## Abstract

**Objective:**

To evaluate the safety and tolerability of multiple intravenous (IV) doses of sifalimumab in adults with moderate-to-severe systemic lupus erythematosus (SLE).

**Methods:**

In this multicenter, double-blind, placebo-controlled, sequential dose-escalation study, patients were randomized 3:1 to receive IV sifalimumab (0.3, 1.0, 3.0, or 10.0 mg/kg) or placebo every 2 weeks to week 26, then followed up for 24 weeks. Safety assessment included recording of treatment-emergent adverse events (AEs) and serious AEs. Pharmacokinetics, immunogenicity, and pharmacodynamics were evaluated, and disease activity was assessed.

**Results:**

Of 161 patients, 121 received sifalimumab (26 received 0.3 mg/kg; 25, 1.0 mg/kg; 27, 3.0 mg/kg; and 43, 10 mg/kg) and 40 received placebo. Patients were predominantly female (95.7%). At baseline, patients had moderate-to-severe disease activity (mean SLE Disease Activity Index score 11.0), and most (75.2%) had a high type I interferon (IFN) gene signature. In the sifalimumab group versus the placebo group, the incidence of ≥1 treatment-emergent AE was 92.6% versus 95.0%, ≥1 serious AE was 22.3% versus 27.5%, and ≥1 infection was 67.8% versus 62.5%; discontinuations due to AEs occurred in 9.1% versus 7.5%, and death occurred in 3.3% (n = 4) versus 2.5% (n = 1). Serum sifalimumab concentrations increased in a linear and dose-proportional manner. Inhibition of the type I IFN gene signature was sustained during treatment in patients with a high baseline signature. No statistically significant differences in clinical activity (SLEDAI and British Isles Lupus Assessment Group score) between sifalimumab and placebo were observed. However, when adjusted for excess burst steroids, SLEDAI change from baseline showed a positive trend over time. A trend toward normal complement C3 or C4 level at week 26 was seen in the sifalimumab groups compared with baseline.

**Conclusion:**

The observed safety/tolerability and clinical activity profile of sifalimumab support its continued clinical development for SLE.

Systemic lupus erythematosus (SLE) is a chronic systemic autoimmune disease with complex pathogenesis and an unpredictable clinical course including flares of disease activity (1–3). It is characterized by the production of autoantibodies, inflammation, and tissue damage in multiple organs from the deposition of immune complexes (1, 2). The consequences of active SLE include organ damage (4), long-term morbidity, and an increased risk of mortality, often from infections and cardiovascular disease (1, 2, 5). Active SLE is also associated with reduced quality of life (6, 7) and high economic burden (8). SLE activity is treated with antimalarials, corticosteroids, and immunosuppressants (3). A biologic treatment targeting B lymphocyte stimulator, belimumab, has recently been approved by the US Food and Drug Administration for use in SLE (9, 10), and a number of other biologic drugs are in development (11, 12). Current treatments often have considerable toxicity and elicit partial or variable responses, so there remains a significant unmet need for treatments with improved efficacy and an acceptable safety profile (12).

The cytokine family of type I interferons (IFNs), and especially the IFNα subtypes, are implicated as important players in SLE pathogenesis (13, 14). Several observations support this. IFNα treatment is sometimes associated with the development of autoantibodies and even SLE-like features (15, 16). In patients with SLE, high type I IFN or IFN-driven chemokine levels are associated with greater disease activity (17–21). Genetic polymorphisms of several components of the IFN signaling pathway have been associated with an increased risk of SLE (21, 22). Furthermore, mice deficient in the IFNα/β receptor have been shown to exhibit reduced signs and symptoms of SLE (23), and the IFNα kinoid vaccine prevents clinical manifestations in a lupus flare model (24). Therefore, IFNα subtypes have been identified as a potential target for drug development in SLE (25).

Sifalimumab (formerly, MEDI-545) is a human anti–IFNα monoclonal antibody that binds to and specifically neutralizes most IFNα subtypes, preventing signaling through the type I IFN receptor (25). In a phase Ia study of patients with SLE, single doses of sifalimumab were shown to have linear, dose-proportional pharmacokinetics (PK) and dose-dependent inhibition of the type I IFN–inducible gene signature. The safety and immunogenicity profile of sifalimumab supported further clinical development (25, 26).

The primary objective of the present study was to evaluate the safety and tolerability of multiple doses of intravenous (IV) sifalimumab in patients with moderate-to-severe SLE. The secondary objectives were to evaluate the PK and immunogenicity of sifalimumab. In addition, the effect of sifalimumab on the expression of type I IFN–inducible genes in the blood and disease activity were evaluated.

## PATIENTS AND METHODS

### Study design

This was a phase Ib, multicenter, randomized, double-blind, placebo-controlled, dose-escalation study of multiple IV doses of sifalimumab in adult patients with SLE (MI-CP152; NCT00482989). The study consisted of a screening period of up to 4 weeks, a 26-week treatment period, and a 24-week followup period. Patients were divided into 4 dose cohorts. Incremental dose escalation occurred following a blinded safety review of data after the twelfth patient reached 6 weeks of exposure.

Patients were categorized by type I IFN–inducible gene signature (low or undetectable versus high) from a panel of 21 type I IFN–inducible genes (25). For each category, treatment was assigned using a central interactive voice response system (block randomization), to avoid a large imbalance of gene signature–positive patients in any one treatment group. The randomization list was generated by United BioSource Corporation. Patients and clinical site staff were blinded with regard to treatment allocation throughout the study. Patients were randomized in a 3:1 ratio to receive either 0.3, 1.0, 3.0, or 10.0 mg/kg sifalimumab or placebo as an IV infusion administered over ≥60 minutes every 2 weeks for a total of 14 doses.

### Patients

Adults age ≥18 years with moderate-to-severe SLE were enrolled in the study. All study participants were required to meet the 4 American College of Rheumatology revised classification criteria for SLE (27, 28), have a Safety of Estrogens in Lupus Erythematosus National Assessment (SELENA) version of the SLE Disease Activity Index (SLEDAI) (29) score of ≥6 or 1 system with a British Isles Lupus Assessment Group (BILAG) score of A or 2 systems with a BILAG score of B (30) at screening, and have a positive antinuclear antibody test (≥1:80 serum dilution) at or prior to screening.

The key exclusion criteria were acute illness (other than SLE) or infection; history of or current severe viral or tuberculosis infection, primary immunodeficiency, or cancer; herpes zoster infection within the past 3 months; abnormal blood test results at screening; recent high (>20 mg/day) or fluctuating doses of oral corticosteroids, antimalarials, or immunosuppressants; B cell–depleting therapies within the past 12 months, treatment with leflunomide in the past 6 months, or any other biologic agent in the past 30 days; treatment with sifalimumab in the past 4 months; or detectable antisifalimumab antibodies at screening. Patients with elevated findings on liver function tests (alanine aminotransferase [ALT] or aspartate aminotransferase [AST] >2 times the upper limit of normal [ULN]) that resulted from liver involvement in SLE in the opinion of the investigator were not excluded from the study.

Concomitant medications were to remain unchanged for 1 month (2 weeks for oral corticosteroids) prior to study start until day 196, apart from burst-and-taper corticosteroids to control SLE flare. Up to two courses, of ≤14 days each, of burst oral steroids of up to 40 mg/day of prednisone or equivalent, or no more than 160 mg/course of intramuscular methylprednisolone or equivalent, were allowed between day 0 and day 126. No burst steroids were allowed on or after day 126, and tapering of burst steroids was to have been completed by day 142. During the followup period after day 196, steroid dosage could be changed as needed.

Written informed consent was obtained from patients before study entry or any study-specific activities were carried out. The study was conducted in accordance with the International Conference on Harmonization Guidance for Good Clinical Practice and the Declaration of Helsinki.

### Assessments

#### Primary end point

The primary end point was the safety and tolerability of sifalimumab. Treatment-emergent adverse events (AEs) and serious AEs (SAEs) and their severity, outcome, and any relationship to the study medication were recorded by the investigator throughout the study. AEs were considered likely to be related to study medication if they were possibly, probably, or definitely related to study medication according to investigator assessment. SLE flare was recorded as an AE only if the organ system involved had been inactive or if disease activity in the organ system involved was considerably worse than it was during the year prior to study entry. Additional safety variables included findings on physical examination, vital signs, electrocardiography, viral cultures and titers, hematology, serum chemistry, and urinalysis.

#### Secondary end points

Blood samples for PK and antisifalimumab antibody assessments were obtained at screening (if the patient had previously received sifalimumab), on day 0, every 2 weeks to week 26 during the treatment period, at the beginning of the followup period (day 185), at weeks 27 and 28, and every 4 weeks during the followup period. Concentrations of sifalimumab in serum samples were measured using a validated enzyme-linked immunosorbent assay method (26). The detection, confirmation, and titer measurement of antisifalimumab antibody were conducted using a validated drug-tolerant, solution-phase, bridging electrochemiluminescence assay at screening (if the patient had previously received sifalimumab), on day 0, at weeks 4, 8, and 16 during the treatment period, and at weeks 28, 34, 42, and 50 during the followup period (see Supplementary text, available on the *Arthritis & Rheumatism* web site at http://onlinelibrary.wiley.com/doi/10.1002/art.37824/abstract).

#### Exploratory end points

Blood samples for measurements of the expression of type I–inducible genes were obtained at screening, on day 0, at weeks 4, 8, and 16 during the treatment period, and at weeks 28, 30, 34, 42, and 50 during the followup period. RNA extracted from the blood was evaluated for the expression of the 21 type I–inducible genes. The median fold change in expression was used as a pharmacodynamic (PD) biomarker (25). In subgroup analyses, 4 of the 21 genes (from baseline specimens) were used to stratify patients into subgroups based on type I IFN gene signature (high or low). As a continuous score, the 4-gene score is 99% correlated with the 21-gene score (see Supplementary Figure 1, available on the *Arthritis & Rheumatism* web site at http://onlinelibrary.wiley.com/doi/10.1002/art.37824/abstract).

Disease activity was measured using 2 instruments: the SELENA–SLEDAI (29) and the BILAG index (31). These were administered at screening, at the beginning and end of the treatment period, and every 4 weeks during the treatment and followup periods by rheumatologists trained in the SELENA–SLEDAI and BILAG assessments. SLE flare was defined as a worsening of the SELENA–SLEDAI score of >3 points from baseline or as a new grade A or B BILAG score in at least 1 of the 8 organ-based systems compared with baseline. Disease activity was also estimated by measuring the levels of complement C3 and C4 in blood.

### Statistical analysis

No formal sample size calculation was performed, since the primary end point was safety and tolerability. A sample size of 148 patients was planned, with 32 patients in dose cohorts for 0.3, 1.0, and 3.0 mg/kg sifalimumab, and 52 patients in the dose cohort for 10.0 mg/kg sifalimumab. Each cohort could be expanded by 4 patients per protocol.

The intent-to-treat (ITT) population (all patients randomized) was used to summarize patient disposition, baseline demographics, and disease characteristics. The modified ITT population, which comprised all patients who were randomized and received study medication, was used for measurements of PD and disease activity. The safety population comprised patients who received any study medication, and the PK population comprised patients in the modified ITT population who had ≥1 valid serum sifalimumab concentration assessment after dosing began.

No formal statistical hypothesis testing was planned for the primary end point. Continuous data were summarized by descriptive statistics, and treatment group comparisons were made using one-way analysis of variance. Categorical data were analyzed by the number and percentage of patients in each category. PK parameters were estimated by noncompartmental analysis using WinNonlin version 5.2 (Pharsight). Serum sifalimumab concentrations were summarized by treatment group and sampling time. The first and last dose peak concentrations, area under the concentration curve within a dosing interval, steady-state clearance, volume of distribution, and terminal-phase half-life were estimated. For disease activity measured by SELENA–SLEDAI and BILAG, the last observation carried forward method was used for missing data. A post hoc analysis of disease activity, measured by SELENA–SLEDAI, was adjusted for burst steroids in excess of that permitted by the protocol. Patients who received excess burst steroids were considered to be nonresponders from the time the burst began.

## RESULTS

### Disposition and baseline characteristics of the patients

**Figure 1:**
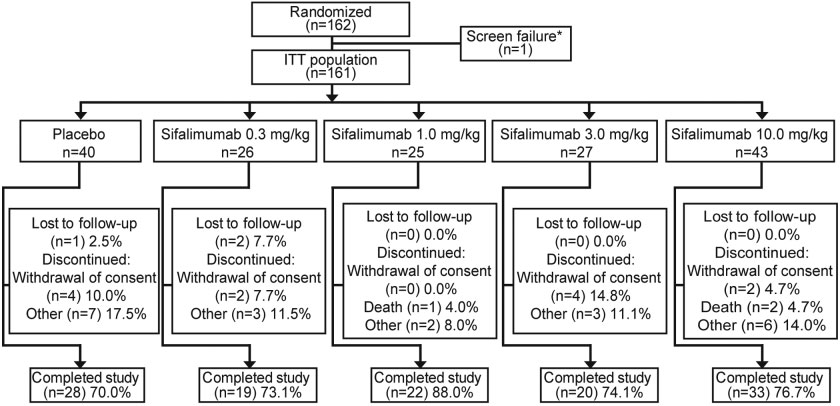
Disposition of the patients. The asterisk indicates a patient who did not meet the inclusion criteria but was inadvertently randomized without receiving the study drug. This patient was excluded from the intent-to-treat (ITT) population.

The study started on March 1, 2008 and was completed on July 20, 2010. Thirty rheumatology centers in 5 countries (Argentina, Brazil, Canada, Chile, and the US) participated. A list of the study investigators and centers is shown in Appendix A. Of the 162 patients who were randomized, 1 patient who did not meet the inclusion criteria was inadvertently randomized but received no study drug, and no data were obtained for this patient. The remaining 161 randomized patients comprised the ITT population, and 122 (75.8%) of these patients completed the study (Figure 1).

With regard to baseline demographic characteristics, the patients were well matched in terms of sex, age, and proportion with a high type I IFN gene signature (Table 1). The combined sifalimumab group had a higher proportion of nonwhite patients than the placebo group (31.4% versus 17.5%) and a higher proportion of black patients (27.3% versus 17.5%). Most patients had a high type I IFN gene signature (75.2%). Baseline disease activity indicated moderate-to-severe SLE despite standard therapy (Table 1).

**Table 1:** Baseline demographic and disease characteristics of the SLE patients (ITT population)*

	Placebo (n = 40)	Combined sifalimumab (n = 121)	Sifalimumab 0.3 mg/kg (n = 26)	Sifalimumab 1.0 mg/kg (n = 25)	Sifalimumab 3.0 mg/kg (n = 27)	Sifalimumab 10.0 mg/kg (n = 43)
Sex, female	39 (97.5)	115 (95.0)	26 (100.0)	23 (92.0)	26 (96.3)	40 (93.0)
Age, mean ± SD, years	44.8 ± 10.9	42.2 ± 11.3	45.0 ± 11.6	41.6 ± 11.6	43.0 ± 11.2	40.3 ± 10.9
High type I IFN†	30 (75.0)	92 (76.0)	17 (65.4)	21 (84.0)	22 (81.5)	32 (74.4)
Race						
White	33 (82.5)	83 (68.6)	16 (61.5)	19 (76.0)	21 (77.8)	27 (62.8)
Black	7 (17.5)	33 (27.3)	10 (38.5)	6 (24.0)	4 (14.8)	13 (30.2)
Asian	0 (0.0)	4 (3.3)	0 (0.0)	0 (0.0)	1 (3.7)	3 (7.0)
Other	0 (0.0)	1 (0.8)	0 (0.0)	0 (0.0)	1 (3.7)	0 (0.0)
North American	29 (72.5)	86 (71.1)	26 (100.0)	14 (56.0)	13 (48.1)	33 (76.7)
Baseline medication						
Oral corticosteroids	26 (65.0)	89 (73.6)	16 (61.5)	20 (80.0)	18 (66.7)	35 (81.4)
Antimalarials	25 (63.0)	86 (71.1)	17 (65.4)	17 (68.0)	20 (74.1)	32 (74.4)
ANA positive	39 (97.5)	121 (100.0)	26 (100.0)	25 (100.0)	27 (100.0)	43 (100.0)
SELENA–SLEDAI score, mean ± SD	10.8 ± 5.0	11.1 ± 5.5	10.7 ± 5.7	10.4 ± 4.2	10.4 ± 4.6	12.2 ± 6.4
≥1 BILAG score of A	11 (27.5)	28 (23.1)	3 (11.5)	2 (8.0)	10 (37.0)	13 (30.2)
≥2 BILAG scores of B and no BILAG scores of A	21 (52.5)	55 (45.5)	15 (57.7)	14 (56.0)	11 (40.7)	15 (34.9)
BILAG domain score of A						
General	1 (2.5)	1 (0.8)	0 (0.0)	0 (0.0)	0 (0.0)	1 (2.3)
Musculoskeletal	5 (12.5)	11 (9.1)	2 (7.7)	0 (0.0)	1 (3.7)	8 (18.6)
Mucocutaneous	5 (12.5)	10 (8.3)	1 (3.8)	1 (4.0)	5 (18.5)	3 (7.0)
Renal	0 (0.0)	7 (5.8)	0 (0.0)	1 (4.0)	3 (11.1)	3 (7.0)
Hematologic	0 (0.0)	1 (0.8)	0 (0.0)	0 (0.0)	0 (0.0)	1 (2.3)
BILAG domain score of B						
General	3 (7.5)	22 (18.2)	7 (26.9)	4 (16.0)	5 (18.5)	6 (14.0)
Musculoskeletal	25 (62.5)	74 (61.2)	18 (69.2)	15 (60.0)	19 (70.4)	22 (51.2)
Mucocutaneous	24 (60.0)	66 (54.5)	17 (65.4)	16 (64.0)	13 (48.1)	20 (46.5)
Renal	5 (12.5)	7 (5.8)	0 (0.0)	0 (0.0)	1 (3.7)	6 (14.0)
Hematologic	5 (12.5)	15 (12.4)	1 (3.8)	3 (12.0)	4 (14.8)	7 (16.3)

* Except where indicated otherwise, values are the number (%) of patients. SLE = systemic lupus erythematosus; ITT = intent to treat; ANA = antinuclear antibody; SELENA–SLEDAI = Safety of Estrogens in Lupus Erythematosus National Assessment version of the SLE Disease Activity Index; BILAG = British Isles Lupus Assessment Group.

† Measured using a 4-gene panel of type I interferon (IFN)–inducible genes.

### Safety and tolerability

The median duration of exposure to study medication was 6.4 months (for both sifalimumab and placebo). The median cumulative amount of sifalimumab received was 322.8 mg in the 0.3 mg/kg group, 899.6 mg in the 1.0 mg/kg group, 2,394.6 mg in the 3.0 mg/kg group, and 7,920.0 mg in the 10.0 mg/kg group. Fewer than half of the patients received all 14 doses of study medication (45.5% in the combined sifalimumab group and 42.5% in the placebo group), although most patients received ≥12 doses of study medication (71.1% in the combined sifalimumab group and 65.0% in the placebo group).

SAEs were reported in 27 patients receiving sifalimumab (n = 60 SAEs) and 11 patients receiving placebo (n = 19 SAEs). The frequencies of SAEs were similar between the 2 treatment groups, with no apparent dose effects across the individual sifalimumab dose groups. The most frequently reported SAEs were musculoskeletal and connective tissue disorders; SLE flare was reported in 4.1% of the patients receiving sifalimumab and 5.0% of those receiving placebo. Seven related SAEs (polyarthritis, pneumonia, ovarian mass, pneumonitis [in 2 patients], cholestasis, and hepatic necrosis) occurred in 6 of the 121 patients (5.0%) in the combined sifalimumab group. In the placebo group, 3 patients (7.5%) experienced 6 SAEs (herpes encephalitis, infection, urinary tract infection, cytomegalovirus infection, pneumonia, and diffuse large B cell lymphoma).

During the treatment and followup period, ≥1 AE was reported in 112 of the 121 patients receiving sifalimumab (92.6%) versus 38 of the 40 patients receiving placebo (95.0%). The AEs reported most frequently during treatment and followup are presented in Table 2. The frequencies of AEs were similar across the different sifalimumab dose groups when compared to placebo. The most frequent treatment-related AEs were urinary tract infection (9.1% in the combined sifalimumab group versus 10.0% in the placebo group), nausea (5.0% in the combined sifalimumab group versus 5.0% in the placebo group), and headache (5.0% in the sifalimumab group versus 2.5% in the placebo group). Most AEs were mild or moderate.

**Table 2:** Treatment-emergent AEs reported in ≥7% of the patients (safety population)*

	Placebo (n = 40)	Combined sifalimumab (n = 121)	Sifalimumab 0.3 mg/kg (n = 26)	Sifalimumab 1.0 mg/kg (n = 25)	Sifalimumab 3.0 mg/kg (n = 27)	Sifalimumab 10.0 mg/kg (n = 43)
SLE flare	9 (22.5)	29 (24.0)	10 (38.5)	5 (20.0)	7 (25.9)	7 (16.3)
Urinary tract infection	10 (25.0)	24 (19.8)	1 (3.8)	4 (16.0)	10 (37.0)	9 (20.9)
Nausea	7 (17.5)	19 (15.7)	5 (19.2)	4 (16.0)	3 (11.1)	7 (16.3)
Hypokalemia	6 (15.0)	18 (14.9)	8 (30.8)	3 (12.0)	1 (3.7)	6 (14.0)
Nasopharyngitis	2 (5.0)	17 (14.0)	1 (3.8)	3 (12.0)	7 (25.9)	6 (14.0)
Diarrhea	7 (17.5)	14 (11.6)	1 (3.8)	5 (20.0)	1 (3.7)	7 (16.3)
Headache	4 (10.0)	14 (11.6)	1 (3.8)	2 (8.0)	4 (14.8)	7 (16.3)
Arthralgia	5 (12.5)	13 (10.7)	8 (30.8)	1 (4.0)	2 (7.4)	2 (4.7)
Upper respiratory tract infection	7 (17.5)	12 (9.9)	2 (7.7)	0 (0.0)	3 (11.1)	7 (16.3)
Vomiting	3 (7.5)	10 (8.3)	4 (15.4)	3 (12.0)	3 (11.1)	0 (0.0)
Dizziness	2 (5.0)	10 (8.3)	1 (3.8)	2 (8.0)	2 (7.4)	5 (11.6)
Fatigue	2 (5.0)	10 (8.3)	2 (7.7)	4 (16.0)	2 (7.4)	2 (4.7)
Decreased hemoglobin	5 (12.5)	9 (7.4)	7 (26.9)	2 (8.0)	0 (0.0)	0 (0.0)
Back pain	3 (7.5)	9 (7.4)	2 (7.7)	2 (8.0)	2 (7.4)	3 (7.0)
Decreased lymphocyte count	3 (7.5)	9 (7.4)	7 (26.9)	2 (8.0)	0 (0.0)	0 (0.0)
Sinusitis	3 (7.5)	9 (7.4)	3 (11.5)	3 (12.0)	1 (3.7)	2 (4.7)

* Values are the number (%) of patients. Adverse events (AEs) are shown in order of descending frequency in the combined sifalimumab group. SLE = systemic lupus erythematosus.

Infections were reported in 82 patients receiving sifalimumab (67.8%) and 25 patients receiving placebo (62.5%). The most frequently reported infections are presented in Table 3. Most infections were mild or moderate.

**Table 3:** Infections reported in ≥3% of the patients (safety population)*

	Placebo (n = 40)	Combined sifalimumab (n = 121)	Sifalimumab 0.3 mg/kg (n = 26)	Sifalimumab 1.0 mg/kg (n = 25)	Sifalimumab 3.0 mg/kg (n = 27)	Sifalimumab 10.0 mg/kg (n = 43)
Urinary tract infection	10 (25.0)	24 (19.8)	1 (3.8)	4 (16.0)	10 (37.0)	9 (20.9)
Nasopharyngitis	2 (5.0)	17 (14.0)	1 (3.8)	3 (12.0)	7 (25.9)	6 (14.0)
Upper respiratory tract infection	7 (17.5)	12 (9.9)	2 (7.7)	0 (0.0)	3 (11.1)	7 (16.3)
Sinusitis	3 (7.5)	9 (7.4)	3 (11.5)	3 (12.0)	1 (3.7)	2 (4.7)
Bronchitis	1 (2.5)	8 (6.6)	4 (15.4)	1 (4.0)	0 (0.0)	3 (7.0)
Viral infection	0 (0.0)	6 (5.0)	1 (3.8)	0 (0.0)	2 (7.4)	3 (7.0)
Influenza	1 (2.5)	5 (4.1)	0 (0.0)	0 (0.0)	0 (0.0)	5 (11.6)
Vaginal infection	1 (2.5)	5 (4.1)	0 (0.0)	2 (8.0)	2 (7.4)	1 (2.3)
Gastroenteritis	1 (2.5)	4 (3.3)	0 (0.0)	0 (0.0)	3 (11.1)	1 (2.3)
Herpes zoster	0 (0.0)	4 (3.3)	0 (0.0)	0 (0.0)	2 (7.4)	2 (4.7)
Pneumonia	2 (5.0)	4 (3.3)	0 (0.0)	1 (4.0)	1 (3.7)	2 (4.7)

* Values are the number (%) of patients. Infections are shown in order of descending frequency in the combined sifalimumab group.

In patients treated with sifalimumab, 11 (9.1%) had ≥1 AE that resulted in permanent discontinuation of study medication. These included 1 patient in the 0.3 mg/kg group (SLE flare and decreased lymphocyte count), 2 patients in the 1.0 mg/kg group (depression, multiorgan failure, and accidental multiple drug overdose; pneumonia), 3 patients in the 3.0 mg/kg group (infusion-related reaction, SLE flare, and bacterial peritonitis), and 5 patients in the 10.0 mg/kg group (SLE flare, cholestasis, headache, dyspnea, pyrexia, contusion, allergic conjunctivitis, abdominal pain, asthenia, B cell lymphoma, respiratory failure, sepsis, pleural effusion, and pneumonia). In the placebo group, 3 patients (7.5%) had ≥1 AE that resulted in permanent discontinuation of study medication (herpes encephalitis and infection, weight increase, and diffuse large B cell lymphoma). Infusion reactions were reported in 1 patient receiving sifalimumab 3.0 mg/kg (grade 1), 2 patients receiving sifalimumab 10.0 mg/kg (grade 2 and grade 3), and 1 patient receiving placebo (grade 1). Three patients developed malignancies, including metastatic chondrosarcoma (1 patient receiving placebo) and B cell lymphoma (1 patient each in the placebo and sifalimumab 10.0 mg/kg groups).

Five deaths occurred, of herpes encephalitis and upper gastrointestinal hemorrhage (in the placebo group), liver failure due to accidental multiple drug overdose (in the sifalimumab 1.0 mg/kg group), congestive heart failure and SLE flare (in the sifalimumab 10.0 mg/kg group), pneumococcal sepsis (in the sifalimumab 10.0 mg/kg group), and cardiorespiratory arrest and septic shock (in the sifalimumab 10.0 mg/kg group). Of these, only the patient who died of pneumococcal sepsis had additional nonfatal AEs (cholestasis and hepatic necrosis), which were considered by the investigator as possibly related to study medication. Although sepsis contributed to the deterioration in health of 2 patients prior to death, the deaths occurred at least 3 months after the last administration of sifalimumab. However, due to the long half-life of sifalimumab, a potential role of the drug in contributing to the infection cannot be excluded. There were other factors or conditions that led to the deaths of these patients, including lymphoma and chemotherapy in the first patient and cholestatic syndrome and liver necrosis in the second patient. Dosing in the 3.0 and 10.0 mg/kg dosing cohorts was paused for 4 weeks after the second death.

Severe (grade 3) hematologic abnormalities of decreased hemoglobin (≥65 to <80 gm/dl) and decreased lymphocyte counts (≥0.2 to <0.5 × 10^3^/μl) were more frequent in the sifalimumab group than the placebo group (5.0% versus 2.5% and 19.0% versus 10.0%, respectively). Severe or very severe hepatic toxicity (defined as grade 3 or 4 increases in AST [>5.0 times the ULN], ALT [>5.0 times the ULN], and/or bilirubin [>3.0 times the ULN]) were seen in 4 patients (3.3%) in the sifalimumab group and no patients in the placebo group. Of these, 2 patients were experiencing an SLE flare, 1 patient had cholestasis and hepatic necrosis, and 1 patient had elevated findings on liver function tests at baseline. No serious abnormalities were seen in urinalysis and vital sign assessments in either the sifalimumab groups or the placebo group.

### Pharmacokinetics and pharmacodynamics

**Figure 2:**
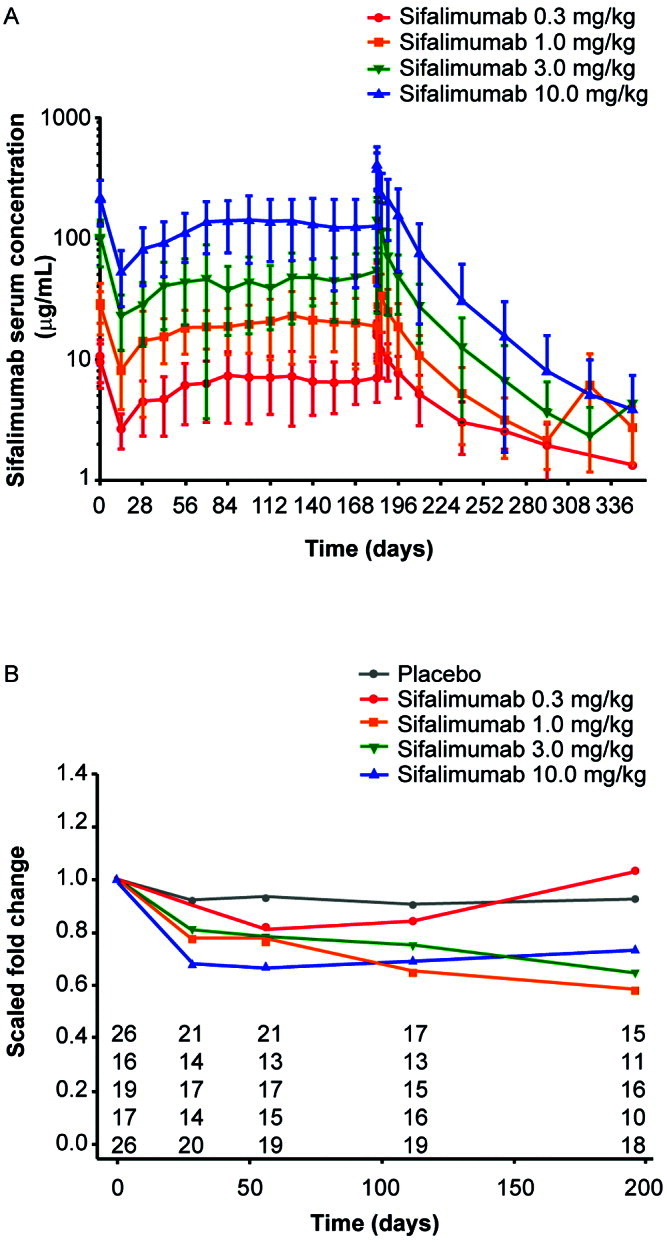
Pharmacokinetics (PK) and pharmacodynamics of sifalimumab over time in patients with systemic lupus erythematosus (PK population). **A,** Serum concentrations of sifalimumab. Patients who missed the last 3 doses were excluded from the analysis. Values are the mean ± SD. **B,** Type I interferon (IFN)–inducible gene signature in whole blood. The type I IFN signature was inhibited by sifalimumab during the treatment phase. Values are the mean fraction of remaining type I IFN signature, using a 21-gene panel, in patients with an increased type I IFN signature at baseline.

Serum concentration–time profiles of sifalimumab following administration of multiple doses of 0.3–10.0 mg/kg and during followup are presented for each dose group in Figure 2A. After multiple doses, serum sifalimumab concentrations accumulated ∼2–3-fold compared with first dose concentrations and reached a steady state by day 84. Steady-state mean maximum concentration and area under the concentration curve within a dosing interval increased dose-proportionally over the dose range 0.3–10.0 mg/kg. The mean steady-state clearance was low (0.19–0.24 liters/day) and similar across the 4 dose groups. The mean volume of distribution at steady state ranged from 5.0 to 6.3 liters, indicating the limited distribution of the monoclonal antibody. Mean terminal half-life was 19.9–29.1 days across the dose groups.

Dose-dependent neutralization of the type I IFN gene signature (21-gene panel) in the blood with sifalimumab treatment was observed in patients who had overexpression of the type I IFN signature at baseline (Figure 2B). Responses to doses of 1.0, 3.0, and 10.0 mg/kg sifalimumab were similar, with a maximum average inhibition of the type I IFN gene signature of 38.7% in the 1.0 mg/kg sifalimumab group.

### Immunogenicity

Overall, 29 of the 121 patients receiving sifalimumab (24.0%) and 1 of the 40 patients receiving placebo (2.5%) had detectable antisifalimumab antibodies at a minimum of 1 visit during the study. The incidence of antisifalimumab antibodies ranged from 11.5% to 29.6% in the sifalimumab groups. Antisifalimumab antibody titers were low (≤80) in most patients (18 of 29). Nine patients had medium titers (>80 to ≤640), and 2 patients had high titers (>640). Detection of antisifalimumab antibodies peaked at ∼16 weeks after the last dose of sifalimumab. Antisifalimumab antibodies had no impact on the PK of sifalimumab in patients who tested positive for antisifalimumab antibodies (data not shown).

### Disease activity

**Figure 3:**
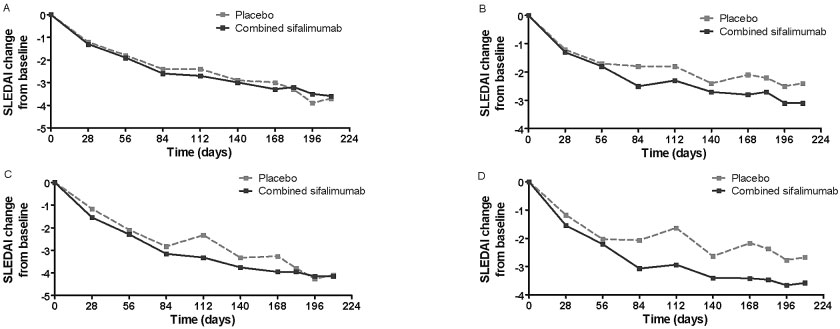
Change from baseline in disease activity, as determined by the Safety of Estrogens in Lupus Erythematosus National Assessment (SELENA) version of the Systemic Lupus Erythematosus Disease Activity Index (SLEDAI) in patients with SLE (modified intent-to-treat population). **A,** Combined sifalimumab group (n = 121) and placebo group (n = 40). **B,** Combined sifalimumab group (n = 121) and placebo group (n = 40) adjusted for burst steroids in excess of that permitted in the protocol. Patients' baseline values were imputed for the value after excess steroid use (dose and duration). **C,** Subgroup of patients with a high type I interferon (IFN)–inducible gene signature (using a 4-gene panel) at baseline (n = 92 in the sifalimumab group and n = 30 in the placebo group). **D,** Subgroup of patients with a high type I IFN–inducible gene signature at baseline adjusted for burst steroids in excess (dose or duration) of that permitted in the protocol (and as described in **B**) (n = 92 in the sifalimumab group and n = 30 in the placebo group). Values are the mean.

The effect of sifalimumab on disease activity was similar to that of placebo, as measured by the mean change from baseline in SELENA–SLEDAI score (Figure 3A). Post hoc analysis adjusting for the use of excess burst corticosteroids (n = 21 patients in the sifalimumab groups and 11 patients in the placebo group) showed a greater mean change from baseline in SELENA–SLEDAI score in sifalimumab-treated patients than in placebo-treated patients (Figure 3B).

Subanalyses of patients with a high type I IFN gene signature at baseline showed a greater mean reduction from baseline in SELENA–SLEDAI score in the combined sifalimumab group compared with the placebo group (Figure 3C). Also, after adjusting for protocol-specified nonallowed use of burst steroids (n = 16 patients in the sifalimumab groups and 8 patients in the placebo group), a clearer trend in SELENA–SLEDAI improvement was observed in patients with a high type I IFN gene signature at baseline in the combined sifalimumab group, as compared with the placebo group (Figure 3D). The mean ± SD change from baseline to week 26 in global BILAG score was similar in the combined sifalimumab group (−2.6 ± 4.5) and the placebo group (−2.4 ± 3.5).

At baseline, 62 of the 121 patients in the combined sifalimumab group (51.2%) and 21 of the 40 patients in the placebo group (52.5%) had abnormally low C3 complement levels (<88 mg/dl for patients ≤65 years old and <82 mg/dl for patients >65 years old), and 64 of the 121 patients in the combined sifalimumab group (52.9%) and 23 of the 40 patients in the placebo group (57.5%) had abnormal C4 complement levels (normal range 16–47 mg/dl). At week 26, fewer patients in the combined sifalimumab group than patients in the placebo group had abnormal complement levels. Abnormal C3 levels were detected in 39 of 96 patients in the sifalimumab group (40.6%) and 13 of 27 patients in the placebo group (48.1%). Abnormal C4 levels were detected in 42 of 96 patients in the sifalimumab group (43.8%) and 16 of 27 patients in the placebo group (59.3%).

## DISCUSSION

In this phase Ib, dose-escalation, safety and tolerability study of multiple doses of IV sifalimumab in adults with moderate-to-severe SLE, sifalimumab demonstrated an acceptable safety and tolerability profile, with a low level of viral and other infections. Similar frequencies of AEs occurred in the combined sifalimumab and placebo groups. The most frequent AEs in the sifalimumab groups were SLE flare, urinary tract infection, nausea, hypokalemia, nasopharyngitis, diarrhea, and headache. AEs were reported at similar frequencies across the dose groups, except for SLE flares, which decreased at higher sifalimumab doses, and headache and viral infection, which were more frequent at higher sifalimumab doses. Discontinuation rates for AEs in the sifalimumab groups (9.1%) and placebo group (7.5%) were similar. Three patients developed malignancies, 1 in the sifalimumab 10.0 mg/kg group and 2 in the placebo group.

Five deaths occurred, 1 in the sifalimumab 1.0 mg/kg group, 3 in the sifalimumab 10.0 mg/kg group, and 1 in the placebo group. There were 2 infections in the sifalimumab 10 mg/kg group, septic shock and pneumococcal sepsis, that resulted in 2 of the deaths more than 3 months after the last dose of sifalimumab. While there were contributing factors that led to the deaths (chemotherapy and lymphoma in 1 patient and hepatic necrosis and cholestatic syndrome in 1 patient), the potential contribution of sifalimumab to the deaths cannot be excluded, due to the long half-life of sifalimumab and the risk assessed with larger numbers of patients and longer periods of observation.

The multiple-dose PK of sifalimumab in this study was linear and dose-proportional over the dose range 0.3–10.0 mg/kg. The low systemic clearance (0.19–0.24 liters/day), small volume of distribution (5.0–6.3 liters), and terminal half-life of 20–29 days were representative of a monoclonal antibody without target-mediated clearance. In the previous phase Ia study of sifalimumab, single-dose PK was also linear and dose-proportional across the dose range tested (1–30 mg/kg). Inhibition of the 21-gene type I IFN signature was dose dependent, and there was a trend toward improvement in disease activity in sifalimumab-treated patients compared with placebo-treated patients (26).

In this study, sifalimumab exhibited a dose-dependent target neutralization as in the phase Ia study described previously (25). In contrast to the early phase Ia study, however, IFN target neutralization was significantly less in this group of SLE patients with moderate-to-severe disease than previously reported in SLE patients with mild disease. The reason for this difference is not clear but may reflect increased contributions of types Iβ and δ IFNs to the target signature in patients with moderate-to-severe SLE. Sifalimumab inhibits most but not all type I IFNα subtypes but does not inhibit β or δ IFNs. The higher incidence of antisifalimumab antibodies, 24.0% in patients receiving sifalimumab and 2.5% in patients receiving placebo, in this multiple-dose study is reflective of the use of a sensitive and drug-tolerant assay for detecting antisifalimumab antibodies. There was no impact of antisifalimumab antibodies on sifalimumab PK in patients who tested positive for antisifalimumab antibodies.

In measurements of disease activity, which were included only as an exploratory end point, the heterogeneous baseline characteristics and the ascending dose design made comparisons between groups difficult, and the small number of patients in each group limited the usefulness of the data. The effect of sifalimumab on disease activity was similar to that of placebo, as assessed by SELENA–SLEDAI and BILAG scores, except in post hoc analyses, in which positive trends were observed in the combined sifalimumab group and the subgroup of patients with a high type I IFN gene signature at baseline, and when these were adjusted for burst steroids in excess of that permitted in the protocol. There was a trend toward normalization of both C3 and C4 complement levels in patients with low levels at baseline, more frequently in the sifalimumab group than the placebo group over time. Complement components (C3 or C4) are considered biomarkers of disease activity in SLE (26).

The heterogeneous nature of SLE (1–3) makes it a prime candidate for individualized treatment strategies to improve patient outcomes. This, in turn, raises the need for biomarkers to identify patients who may benefit from anti-IFNα therapies. The 21-gene type I IFN signature has been successfully used as a PD marker (25, 26, 32). A 4-gene subset of these 21 genes has subsequently been identified as a potential predictive marker to identify patients who may respond to sifalimumab treatment (20, 33). In the post hoc analyses of this study, the 4-gene signature was used to stratify patients into groups of those with high and those with low type I IFN activity, with a prevalence of high type I IFN activity of ∼75% in patients with moderate-to-severe SLE. Inhibition of the expression of the type I IFN gene signature by sifalimumab is indicative of its inhibition of type I IFN signaling by blockade of IFNα. Incomplete suppression of the type I IFN gene signature both in this study and in the earlier phase I study (26) suggests that other type I IFNs in SLE may contribute to the activation of the type I IFN pathway. This hypothesis is being investigated.

Limitations of this study include the relatively small sample size. Therefore, results should be interpreted with caution, especially for data on disease activity, since this was an exploratory end point. It should be noted that the cohorts had variable enrollment by country and site, which may have introduced bias. For example, the sifalimumab 0.3 mg/kg cohort was enrolled entirely from the US, while the sifalimumab 3.0 mg/kg cohort was less than half US-based. Since patients in the placebo group were randomized with each dosing cohort, this also meant that the placebo group contained variations by geographic origin. A further geographic difference was noted with regard to type I IFN gene signature. A higher proportion of patients enrolled in South America (91.3%; n = 46) had a high type I IFN gene signature than those enrolled in North America (69.6%; n = 115).

A 4-week interruption in study drug administration while the death of 1 study patient was investigated occurred late in the study, and this meant that the interruption of dosing affected chiefly the 10.0 mg/kg cohort. The influence of changes in background medication, such as burst-and-taper courses of corticosteroids, on disease activity measures was another limitation of the study design. Finally, the heterogeneous baseline characteristics of this study population make between-group comparisons difficult. This is, however, a common difficulty in conducting clinical studies in SLE, where patients have highly variable disease characteristics in terms of organs affected, severity of organ damage, disease activity, and frequency and severity of SLE flares, and may also have AEs induced by standard therapy (29, 34–36).

In conclusion, at multiple IV doses, sifalimumab appears to have an adequate safety and tolerability profile in adults with moderate-to-severe SLE, supporting the continued clinical development of sifalimumab in this disease. A currently ongoing larger phase IIb, double-blind, placebo-controlled, parallel-design study is under the observation of an independent data safety monitoring board to further characterize the safety profile.

## AUTHOR CONTRIBUTIONS

All authors were involved in drafting the article or revising it critically for important intellectual content, and all authors approved the final version to be published. Dr. Petri had full access to all of the data in the study and takes responsibility for the integrity of the data and the accuracy of the data analysis.

**Study conception and design.** Petri, Wallace, Spindler, Chindalore, Mysler, Neuwelt, Robbie, White, Yao, Ethgen.

**Acquisition of data.** Petri, Wallace, Spindler, Chindalore, Kalunian, Mysler, Neuwelt, Greth.

**Analysis and interpretation of data.** Petri, Wallace, Mysler, Neuwelt, Robbie, White, Higgs, Yao, Wang, Ethgen, Greth.

## ROLE OF THE STUDY SPONSOR

MedImmune, LLC was responsible for the study design, data monitoring and collection, data analysis, and data interpretation. All authors, including those who are employees of the sponsor, contributed to the interpretation of the data and writing of the article, and were responsible for the decision to submit it for publication. The corresponding author had full access to all the study data and had final responsibility for the decision to submit the article for publication. Medical writing and editorial assistance was provided by Sally Cotterill, PhD, from QXV Communications, Macclesfield, UK.

## APPENDIX A: STUDY INVESTIGATORS

The following investigators contributed to the study: in Brazil, Sebastiao C. Radominski (Centro de Estudos em Terapias Inovadoras LTDA, Curitiba), Morton A. Scheinberg (Associação de Assitência à Criança Deficiente, São Paulo), and Cristiano A. Zerbini (Centro Paulista de Investigação LTDA, São Paulo); in Canada, Christine A. Peschken (Arthritis Centre, Winnipeg, Manitoba); in Chile, Imgadt A. Goecke (Prosalud y Cia Ltda, Santiago) and Pedro Miranda (Centro de Estudios Reumatológicos, Santiago); in the US, Anca D. Askanase (New York University–Hospital for Joint Diseases, New York, NY), Seth M. Berney (Louisiana State University Health Sciences Center, Shreveport), Stephen A. Bookbinder (Ocala Rheumatology Research Center, Ocala, FL), Michael Burnette (Tampa Medical Group, Tampa, FL), Thomas D. Geppert (Metroplex Clinical Research Center, Dallas, TX), John A. Goldman (Atlanta Center for Clinical Research, Atlanta, GA), Kyriakos A. Kirou (Hospital for Special Surgery, New York, NY), Steven J. Klein (Osteoporosis and Clinical Trial Center, Cumberland, MD), Robert W. Levin (Clinical Research of West Florida, Clearwater), Meggan C. Mackay (Feinstein Institute for Medical Research, Manhasset, NY), Robert T. Matheson (Oregon Medical Research Center, Portland), Andreas Reimold (University of Texas, Southwestern Medical Center, Dallas), Georgia C. Roane (Rheumatology Associates, Charleston, SC), Yvonne Sherrer (Centre for Rheumatology, Immunology, and Arthritis, Fort Lauderdale, FL), Timothy J. Swartz (Rheumatology, P.C., Kalamazoo, MI), Edward L. Treadwell (East Carolina University, Greenville, NC), and Frank R. Wellborne (Houston Institute for Clinical Research, Houston, TX).
