# Supplementary material for: Sifalimumab, a Human Anti–Interferon-α Monoclonal Antibody, in Systemic Lupus Erythematosus: A Phase I Randomized, Controlled, Dose-Escalation Study: A Phase I Randomized, Controlled, Dose-Escalation Study
Source: Arthritis Rheum. 2013 Mar 28;65(4):1011–21. doi: 10.1002/art.37824 (PMC3654174; doi:10.1002/art.37824)
Supplement: Supplementary file 1 [file art0065-1011-SD1.doc]

# Supplemental information

Measurement of Anti-sifalimumab Antibodies (ADA) in Human Serum Samples Using a Validated, Sensitive Electrochemiluminescent Assay (ECLA).

In the ECLA for ADA, biotinylated sifalimumab and ruthenylated sifalimumab were incubated overnight with human serum samples that were collected prior to sifalumumab administration on day 1 and at various time points throughout the clinical trial. Subsequently, samples were incubated on a streptavidin-coated Mesoscale Discovery (MSD) plate for capture of the ADA-bridged complexes. The plate was then washed to remove unbound materials, read buffer was added and the plate was placed on the MSD Sector™ Imager for generation and measurement of ECL response. The signal intensity was proportional to the amount of ADA present in the sample.

The  presence of ADA was determined relative to a cutoff ECL value that was calculated for each plate as the mean response of 6-8 wells of the negative control multiplied by a 1.18 cut point factor. The 1.18 cut point factor was established during validation from 200 measurements of serum samples obtained from 50 normal individuals and was statistically determined to provide a 5% false positive rate. Samples that measured at or above the cut point ECL value were considered as potential positives for ADA and were retested in a confirmatory (specificity) assay, both in the absence and presence of excess (300 μg/mL) sifalimumab. The confirmatory cut point was established during method validation using the percent inhibition measurements of the above-mentioned normal samples tested both in the absence and presence of excess sifalimumab. The confirmatory cut point was statistically determined to provide a 0.1% false positive rate. Confirmed positive samples were then measured in a titer assay. Titers were performed by serially diluting samples with negative control serum and were reported as the reciprocal of the highest 1:2 dilution (over the 1:10 minimum required sample dilution) that measured positive in the assay.
