# Supplementary material for: Sifalimumab, a Human Anti–Interferon-α Monoclonal Antibody, in Systemic Lupus Erythematosus: A Phase I Randomized, Controlled, Dose-Escalation Study: A Phase I Randomized, Controlled, Dose-Escalation Study
Source: Arthritis Rheum. 2013 Mar 28;65(4):1011–21. doi: 10.1002/art.37824 (PMC3654174; doi:10.1002/art.37824)
Supplement: Supplementary file 2 [file art0065-1011-SD2.doc]

# **Supplemental Figure: Correlation between 21 gene type I IFN signature scores and 4 gene type I IFN signature scores for 262 SLE patients**

**
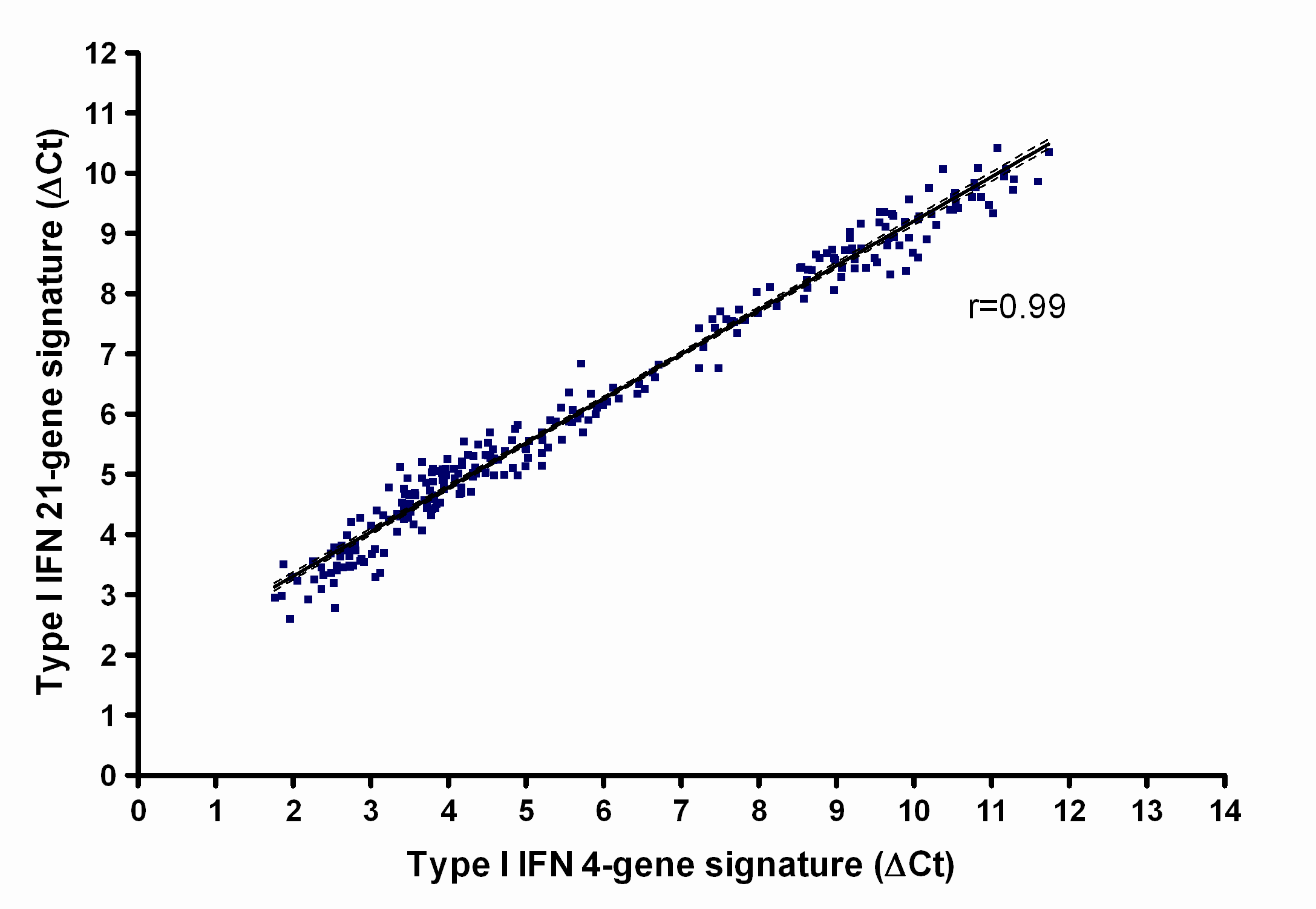
**
